# Decrease in Bodily Weight after Epileptic Attacks

**Published:** 1880-06

**Authors:** 


DECREASE IN BODILY WEIGHT AFTER EPILEPTIC ATTACKS.

P. KOWABEWSKI, Medicinskoje Obosrenje, October, 1879, (abstract in St. Petersburg *Med. Wochenschr*). By regular daily weighing of epileptics, during their atttacks as well as in the intervals, the author comes to the following conclusions:

1. In all epileptics and in all kinds of epilepsy there is a decrease of body weight after each attack, corresponding to its duration and intensity.

2. In old cases in which the attacks were very frequent, and the organism has become accustomed to them, the decrease is very slight after each attack (½ pound Russian); in recent cases, on the other hand, in which the attacks occur as yet but seldom there is a notable decrease (3-12 pound) after each attack.

3. When several attacks follow one another in quick succession, the greatest loss of weight follows the first one; that after the succeeding ones being very slight.

4. The greatest loss of weight in all forms of motor or somatic epilepsy is found after epileptic convulsions (grand mal) equaling some times 12 pounds after a single attack; it is very much less after epileptic vertigos. But the greatest loss of weight is met with in psychic epilepsy, in which case it may equal one-fourth of the whole body weight.

The recovery of body weight after the attacks follows very quickly, requiring only a few days.—*Journal of Nervous and Mental Diseases.*

